# The Ivy Street Hospital

**Published:** 1884-08-20

**Authors:** 


### THE IVY STREET HOSPITAL.

THE IVY STREET HOSPITAL, on the grounds of the Southern Medical College, and the medical management of which is in charge of that Institution, has just undergone papering and calcimining, and is now well supplied with additional bedding, furniture, etc. We invite the attention of our readers to the following card extract from the Announcement of the Southern Medical College, which has just been issued:

"*To the Medical Profession*:

"We invite the attention of members of the Profession, residing out of the city, to the fact that we are prepared to receive and to treat two classes of patients in the Ivy Street Hospital, and that Physicians are desired to send us any patients whom they do not find it convenient to treat at home.

"First, Pauper Patients, white or colored, male or female, sent and paid for by the authorities of any county, at rates as follows, to-wit:

| | |
|---|---|
| Board and nursing per month | $25 00 |
| Board and nursing per week | 6 00 |
| Medical treatment free of charge. | |

"Second, Pay Patients at the following rates:

| | |
|---|---|
| Board and nursing per month | 25 00 |
| Medical treatment at such moderate rates as may be agreed upon between the patient and the physician who may be preferred by the patient, or appointed to treat the case. | |

"The building is well arranged, consisting of three stories, with several out buildings, all arranged so as to accommodate white and colored, male and female patients in separate departments. There are many private rooms, well furnished and comfortable, where patients can be as private as they desire. In the building there are two resident physicians, with a matron and nurses, who do all in their power to contribute to the good and comfort of the patients. The Hospital is only two blocks from Union Depot, and easily accessible, though removed from the noise of the city. When notified, one of the physicians will meet patients at the depot.

"Applicants should first address Dr. Thomas S. Powell, General Supervisor of Ivy Street Hospital."

